# The Metabolic Screening in Wounded Scales of *Hippeastrum* × *hybridum* Hort. Bulbs

**DOI:** 10.3390/ijms26189179

**Published:** 2025-09-19

**Authors:** Wiesław Wiczkowski, Lesław B. Lahuta, Dorota Szawara-Nowak, Karolina Stałanowska, Marian Saniewski, Agnieszka Marasek-Ciołakowska, Justyna Góraj-Koniarska, Marcin Horbowicz

**Affiliations:** 1 Institute of Animal Reproduction and Food Research of the Polish Academy of Sciences, Biotransformation and Bioavailability of Phytochemicals Team, Trylińskiego 18, 10-683 Olsztyn, Poland; w.wiczkowski@pan.olsztyn.pl (W.W.); d.szawara-nowak@pan.olsztyn.pl (D.S.-N.); 2 Department of Plant Physiology, Genetics and Biotechnology, University of Warmia and Mazury, Oczapowskiego 1A, 10-719 Olsztyn, Poland; lahuta@uwm.edu.pl (L.B.L.); karolina.stalanowska@uwm.edu.pl (K.S.); 3 The National Institute of Horticultural Research, Konstytucji 3 Maja 1/3, 96-100 Skierniewice, Poland; marian.saniewski@inhort.pl (M.S.); agnieszka.marasek@inhort.pl (A.M.-C.); justyna.goraj@inhort.pl (J.G.-K.)

**Keywords:** *Hippeastrum*, amaryllis, bulb scales, wounding stress, primary metabolites, secondary metabolites

## Abstract

The aim of this study was to determine changes in primary and secondary metabolites after four days of storage of mechanically wounded *Hippeastrum* × *hybridum* Hort. (amaryllis) bulbs. Mechanically wounded scales of amaryllis bulbs stored for four days change color from white to orange-red, which is the plant’s protective response to stress caused by damage. During this process, changes in the content of primary metabolites (carbohydrates, amino acids, organic acids) and secondary metabolites (flavonoids, phenolic acids, anthocyanins) were analyzed. The resulting color of *Hippeastrum* scales is due to the presence of several pigmented phenolic compounds: flavonoids, phenolic acids and anthocyanins. In particular, the increase in anthocyanin and luteolin content in stored scales probably affected the intensity of their color. The decrease in l-phenylalanine content in the *Hippeastrum* scales to trace levels indicates high intensity of phenolic compound biosynthesis. The increased content of 4-coumaric, ferulic and sinapic acids suggests that the lignification process also occurs. Moreover, the observed significant decrease in glucose, galactose, and sucrose levels indicates intense respiration processes and biosynthesis of various metabolites, which may contribute to counteracting wound-related stress. Mechanical wounding and storage of wounded *Hippeastrum* bulbs for several days may be a simple way to improve the quality of pharmaceutical products manufactured from these bulbs, but this requires further investigations.

## 1. Introduction

Wounding or mechanical damage is the loss of physical integrity of a plant’s cells, tissues or organs. Wounding poses a serious threat to plant tissues which can open the way for pathogen attack or the result of pest feeding, while weather conditions are responsible for mechanical damage [1]. As a result, wounding can lead to water loss and the occurrence of drought stress. Plant defense against wounding stress can be divided into synthesis of secondary metabolites and specific proteins [2]. Proteins encoded by wound-induced genes may be involved (1) in the production of toxic compounds or reduce the digestibility of plant tissue for herbivores; (2) in the activation of wound defence pathways; or (3) in the repair of damaged tissue by adapting the plant’s metabolism to the increased demand for energy and repair components [1,3]. In response to mechanical injury, plants can lead to changes in metabolite content by synthesising and accumulating defense compounds [4,5,6]. Such compounds are various polyphenols and alkaloids, which act as antimicrobial and antifungals agents [7,8].

The tissues of Amaryllidaceae, to which *Hippeastrum* × *hybridum* Hort. (amaryllis) belongs, contain numerous bioactive compounds, including specific alkaloids found exclusively in this plant subfamily [8]. Therefore amaryllis species are a valuable source of bioactive compounds with therapeutic applications [9,10,11]. A recently published paper showed that 48 alkaloids with potential pharmacological significance were identified in *Hippeastrum* species native to Bolivia [9]. Lycorine and homolycorine alkaloids were dominant in *H. chionedyanthum* and *H. haywardii* species, and these species have particularly high levels of lycorine, which is a promising anti-cancer compound [9]. In turn, the species *H. evansiarum* and *H. mollevillquense* contained significant amounts of galantamine-type alkaloids, which are important in the treatment of Alzheimer’s disease [9].

Besides a large group of alkaloids, *Hippeastrum* species also contain many phenolic compounds that are also medically important [10,11]. The phenolic compounds present in *Hippeastrum* also have anti-fungal properties in plant diseases [12,13]. One of the most common diseases that amaryllis plants suffer from is red spotting caused by infection with the fungus *Phoma narcissi* Aderh. (syn. *Stagonospora curtisii* (Berk.) Sacc.) [12]. *Phoma narcissi* is a worldwide-known pathogen of *Hippeastrum*, *Narcissus*, *Hymenocallis* and various species of Amaryllidaceae causing red or reddish-brown spots on various organs [13]. The plant’s defense against the threat of *Phoma narcissi* involves producing a mixture of orange-colored chalcones and flavanes, which can then be oxidised to red dimers or polymers [13,14]. These compounds prevent penetration of injured tissues by *Phoma narcissi*, *Botrytis cinarea*, *Fusarium oxysporum* and *Phoma poolensis* [13,15,16,17,18]. It has been shown that compounds with antifungal properties are present in the red-dyed tissues. As the content of these compounds increases, the infection caused by *Phoma narcissi* has been inhibited [15].

Detailed analysis showed that this pigment, which is absent in unwounded tissue, is a mixture of one orange chalcone and three colorless flavans [14]. Colorless flavans can be oxidised to red dimers or polymers. The light absorption maximum of the compounds from the reddish colored organs extracted with methanol/HCl (99:1) was 476 nm indicating the absence or low content of anthocyanins among them [12,14]. In contrast, the maximum absorption of the red compounds in the amaryllis cv. Red Lion flower was 516 nm, indicating the presence of anthocyanins.

The red color formation reaction observed in wounded *Hippeastrum* scales was accompanied by an increase in methyl jasmonate (JA-Me) content, while reducing the JA-Me content lowered the plant’s ability to produce red pigment [19]. Similarly, mechanical wounding or JA-Me application induced activity of the chalcone synthase in white spruces needles, catalyzing the first step in flavonoid biosynthesis [4].

Phenolic compounds ubiquitous in plant tissues are generally divided into two main groups: flavonoids and non-flavonoids [20]. Phenolic compounds are secondary metabolites that do not directly affect plant growth and development, but they regulate metabolic pathways through signal transduction [21]. These compounds protect plants from disease/damage and also affect the aroma and flavour and especially the colour of the plant [22,23]. The biosynthesis of phenolic compounds is dependent on the availability of l-phenylalanine (l-phe), the main amino acid used as a precursor for the biosynthesis of phenylpropanoids, and l-phe is biosynthesized by the shikimic acid pathway [24]. Abiotic stress in plants induces the biosynthesis and accumulation of polyphenols, which help them adapt to stress conditions [25]. Polymers formed from phenylpropanoids, such as lignin, suberin, and tannins, contribute to the stability and resistance of plants to mechanical or environmental damage, such as drought and wounding [26,27,28,29,30].

Flavonoids are closely associated with plant defense against various stresses [31]. It is known that certain flavonoids induced in plants during pathogen attacks or after mechanical injury play an important role in plant resistance against microbial infections [32]. Flavonoid accumulation is one of the main responses of *Dracaena cochinchinensis* [33] and *Marchantia polymorpha* L. [34] to wound stress. Also, in mechanically crushed red onion peel, the total flavonoid content was twice as high as compared to the control. Among these flavonoids, the highest increase in content in crushed onion peel was for quercetin and quercetin 4′-*O*-glucopyranoside [35]. In addition, the antioxidant activity and total phenolic content were increased by very fine milling of the red rice grains [36].

The aim of this study was to determine changes in primary and secondary metabolites after four-day storage of mechanically wounded scales of *Hippeastrum* bulbs. The main purpose was to determine the content of compounds that may affect the color appearing after storage of wounded amaryllis bulbs. This study is a continuation of our earlier research [12,13,15,16,17,18,19]. Amaryllidaceae tissues are potentially important as a source of compounds with pharmacological significance. Therefore, the study also aimed to determine how mechanical wounding of *Hippeastrum* bulbs affects the ingredients they contain.

## 2. Results

**Figure 1:**
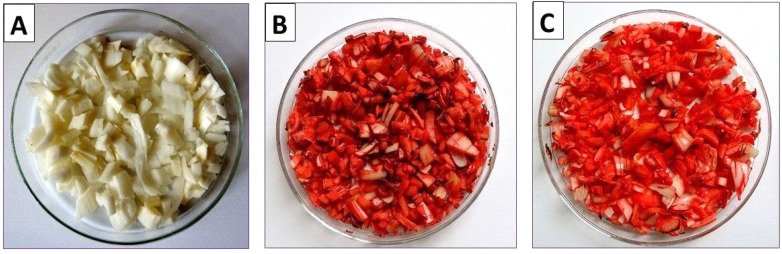
Appearance of *Hippeastrum* (amaryllis) bulb scales immediately after mechanical wounding (**A**) and after wounding and 4 days of storage: in daylight (**B**) and in darkness (**C**).

Immediately after mechanical wounding, the scales were white with a slight grayish tint (Figure 1A), and after 4 days of storage in daylight (Figure 1B), they were intensely red-orange. However, scales that were cut and stored in the dark had a lighter orange-red color than those stored in daylight (Figure 1C).

**Figure 2:**
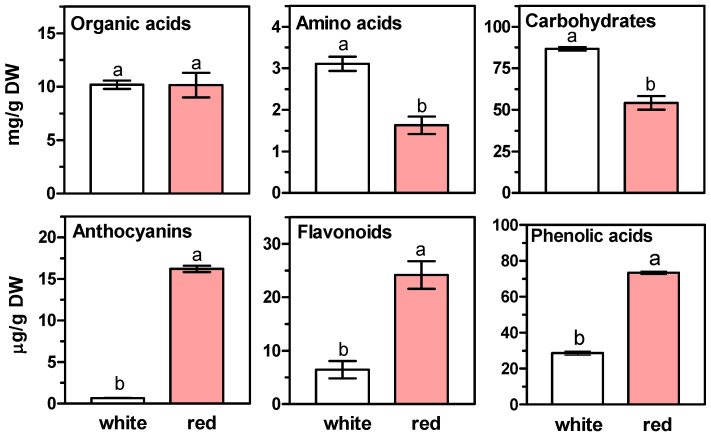
Total contents of organic acids, amino acids, carbohydrates, anthocyanins, flavonoids, and phenolic acids in scales of *Hippeastrum* (amaryllis) bulbs immediately after mechanical wounding (white) and after wounding and 4 days of storage under daylight conditions (red). Bar results (means ± SD) marked with the same letter do not differ at the significance level of *p* < 0.05 according to Duncan’s test.

In mechanically cut scales of amaryllis bulb, which were then stored in daylight, clear changes were observed in most of the secondary metabolites. In these tissues, a significant increase in the content of total phenolic compounds, i.e., phenolic acids, anthocyanins and flavonoids, was observed after four days of storage (Figure 2). A particularly large increase in content occurred in the case of anthocyanins. At the same time, a decrease in the total content of carbohydrates and amino acids was noted, while the content of organic acids remained unchanged.

The following phenolic acids were identified in the scales of the *Hippeastrum* bulbs: ferulic, 4-coumaric, protocatechuic, caffeic, caftaric, ferulic, ellagic and synapic (Table 1).

The phenolic acid that was quantitatively predominant was ferulic acid, whose total content was approximately 80% in freshly cut scales of amaryllis bulbs. After storage, the total content of this acid was about 70% of all acids. Most of the phenolic acids (ferulic, 4-coumaric, caffeic, and synapic) occurred in ester form. Ellagic acid, on the other hand, occurred mainly in the form of aglycone. Four-day storage of the cutting scales resulted in a significant increase in the content of the particular forms of phenolic acids and their total contents (Table 1). Among the acids and their forms present, the highest 5-fold increase after 4 days of storage occurred for caffeic acid esters and a 4-fold increase for 4-coumaric acid esters as well as the ellagic acid. Cutting of amaryllis bulbs and storing them for 4 days had no significant effect on the content of phenolic acid glycosides.

The flavonoid content in the scales of amaryllis bulb was significantly lower than the phenolic acid content (Table 2).

Flavonoids were present in these tissues mainly in the form of aglycones, with the exception of catechin, which was present as glycosides. The content of particular flavonoids increased after 4 days of storage of the wounded scales compared to the content immediately after wounding. The exception was catechin, whose glycoside content decreased, but not significantly. In wounded and stored amaryllis scales, the main flavonoid in terms of quantity was luteolin aglycone. The content of this flavonoid before storage was less than 0.1 μg/g DW, while after storage it increased to over 12 μg/g DW (Table 2). A similar great increase in content occurred in the case of naringenin aglycone, from 0.02 μg/g DW after cutting to 5.70 μg/g DW after 4 days of storage.

Eight anthocyanins were detected in the scales of amaryllis bulbs: cyanidin and pelargonidin aglycones, cyanidin, delphinidin, and pelargonidin monoglucosides, and cyanidin, peonidin, and delphinidin diglucosides (Table 3).

Four days of storage of the wounded scales resulted in a rapid increase in anthocyanin content, especially cyanidin and pelargonidin aglycones. After storage, the main quantitatively anthocyanins were cyanidin and pelargonidin aglycones, which accounted for more than half of their total content.

In fresh scales of amaryllis bulb, malic acid and citric acid were the quantitatively dominant organic acids (Table 4). After wounding of the scales and their subsequent storage, the organic acid content did not change significantly, with the exception of acetic acid, whose level decreased slightly. After 4 days storage of scales, the content of eight of the twelve amino acids (valine, serine, leucine, isoleucine, proline, l-phenylalanine, aspartic acid, and γ-aminobutyric acid) decreased (Table 4). Particularly large decreases in content occurred for serine, aspartic acid and l-phenylalanine.

The content of most carbohydrates present in the wounded scales of amaryllis bulb decreased after 4 days of storage. This was the case for galactose, glucose, sucrose, and kestose (Table 4). The sucrose and galactose contents were twice as low, while the glucose content was two and a half times lower after storing cut scales compared to those before storage. In contrast, fructose and *myo*-inositol contents did not change significantly during the time of storage.

## 3. Discussion

Plant wounding can be caused by pest feeding, as well as by weather conditions or human activity, leading to the loss of physical integrity of plant cells, tissues, or organs [1]. As a result, this process can lead to water loss and the occurrence of drought stress [2,3]. Plants can respond to wounding and infection by synthesizing and accumulating defense compounds [4,5,6].

The production of colorful compounds in mechanically wounded *Hippeastrum* bulbs is a response to the threat caused by tissue injury [13,14,15,16,17,18,19]. The compounds produced at that time help prevent *Phoma narcissi* fungal disease [17]. Detailed analysis showed that this pigment, which is absent in unwounded tissue, contains a mixture of flavonoids. According to Wink and Lehmann [14] this pigment is a mixture of an orange chalcone: 3,2′4′-trihydroxy-4-methoxychalcone and three colourless flavans: 7,4′-dihydroxy-8-methylflavan, 7,3′-dihydroxy-4′-methoxyflavan and 7-hydroxy-3′,4′-methylene dioxyflavan. Our current investigation demonstrated that the content of almost all phenolic compounds present in the examined tissues significantly increased (Figure 2; Table 1, Table 2 and Table 3). At the same time, the content of l-phenylalanine in wounded amaryllis bulbs decreased to trace levels, which may indicate its use in the intensive biosynthesis of these phenolic compounds (Table 4).

Plant wounding induced the transcription of major enzymes of the phenylpropanoid pathway, which are critical for the synthesis of phenolic compounds and the biosynthesis of suberin, lignin, and flavonoids [4,29]. Among the secondary metabolites, the 3-*O*-rhamnosides of quercetin and myricetin content were higher in wounded *Catharanthus roseus* roots than in the control [37]. In addition, in the wounded leaves the levels of gallic acid, salicylic acid and daidzein were increased [37]. However, in *Arabidopsis* leaves, the salicylic acid content increased rapidly after wounding, and 24 h after wounding, there was an increase in the content of salicylic acid glucoside and its ester derivative [36].

The results of our study indicate that biosynthesis and significant accumulation of flavonoids and phenolic acids are important components of the response to wound stress, confirming earlier reports [4,29,36,37]. Also, in wounding carrot roots and cassava roots there was an increased accumulation of flavonoids caused by the up-regulation of genes involved in their biosynthesis [38,39]. Moreover, wounding induced the phenylpropanoid pathway and biosynthesis of luteolin, apigenin and isoricardin C in *Marchantia polymorpha* L., while α-aminooxy-β-phenylpropionic acid, an inhibitor of phenylalanine ammonialyase activity, inhibited production of the mentioned phenolic compounds [33].

The more than 200-fold increase in luteolin content demonstrated in our study appears to be a specific indicator of the response of the *Hippeastrum* scales to wound stress (Table 2). The increase in luteolin content relates almost exclusively to its aglycone, which may indicate its involvement in the coloring of wounded scales. Luteolin has an intense yellow color, so a significant increase in its content in wounded amaryllis scales may affect their color. There is little data on the occurrence and content of flavonoids in *Hipeastrum* tissues. The derivatives of 7-hydroxy flavanone and the flavonol rutin were identified in *Hippeastrum vittatun* [10] and in *Hippeastrum stapfianum* [40], and recently, the flavonol quercetin 3-O-rhamnose was identified in *Hippeastrum stapfianum* [41]. However, there is no data available on the flavonoid content in *Hippeastrum x hybridum* bulb tissues.

In addition, increased content of phenolic acids observed in the scales of amaryllis can also affect the intensity and tone of their color because these acids are usually yellow, creamy or pale yellow. However, wounded scales contained mainly phenolic acid esters rather than their aglycones. The available literature has no data on the presence and content of phenolic acids *Hippeastrum* × *hybr*. Hort. bulb tissues.

It is widely known that light is the main factor influencing anthocyanin biosynthesis in plants [42,43]. Light affects this accumulation by regulating the expression of genes in the anthocyanin synthesis pathway [44,45]. Recently published data show that storing cut lily bulbs in ambient light caused them to turn purple-red, a color resulting from the accumulation of anthocyanins [46]. The authors suggest that this phenomenon is the result of increased activity of key enzymes and gene expression in the anthocyanin synthesis pathway. The color and quality of hyacinth bulbs were also affected by the light conditions during their storage [47]. Under the influence of blue light, the bulbs were smaller, but they were colored by the presence of anthocyanins and chlorophyll. Red light, on the other hand, did not cause any coloring of the hyacinth bulbs [47].

The results of our study show that anthocyanin content in freshly cut scales of the *Hippeastrum* bulb was very low (Table 3). Analyses of anthocyanin content in the underground parts of bulbous plants are not performed, which does not mean that they are unnecessary. This is demonstrated by the results of our research, which clearly show that after four days of storage, the wounded scales had a significant increase in the content of all detected anthocyanins. It seems that storage process in the presence of daylight is responsible for the increase in the content of these pigments. The color of the wounded scales stored in darkness was lighter than that of the bulbs stored in the daylight conditions (Figure 1). Cyanidin has a characteristic red color, and its glucoside is also red, while pelargonidin is orange. Therefore, it is likely that the greatly increased anthocyanin content led to a darker or more intense color of the wounded and stored scales of amaryllis bulbs. Unfortunately, no analysis was carried out on the presence and content of anthocyanins in the tissues of the scales stored in darkness.

In soybean leaves, rapid accumulation of GABA was found in response to mechanical stimulation or damage [48]. However, the results of our study showed that the GABA content in wounded and stored scales of *Hippeastrum* bulb decreased. The marked decrease in glucose, galactose and sucrose levels in wounded scales of the amaryllis bulbs observed in our study indicates a high intensity of respiratory processes. These processes are a source of energy and many important metabolites that may be involved in counteracting wound stress [1,2,3]. These metabolites are essential to counteracting the risk of disease and water loss associated with stress caused by wounding. The demonstrated lack of reduction in fructose content may indicate the intensity of the release of this carbohydrate as a result of hydrolysis of 1-kestose (a di-fructose derivative of glucose), or other fructans that could not be identified and determined by the method applied. It has been shown that the use of starch and sucrose may be the material basis for the response to mechanical damage in *Aquilaria sinensis* [49]. However, in wounded leaves and roots of *Catharanthus roseus*, the relative sugar content appeared to be higher compared to the unwounded control [37].

Stress induced by wounding triggers the repair of plant tissue damage, in which lignin plays a role. The primary substrates for lignin biosynthesis are monosaccharides or closely related compounds [50,51]. The lignification process involves the polymerization of monolignols, i.e., 4-coumaryl, coniferyl, and sinapyl alcohols [49]. The monolignol biosynthesis is common to the general phenylpropanoid pathway. Lignin biosynthesis uses l-phenylalanine for the biosynthesis of 4-coumaric acid and CoA esters of 4-coumaric, ferulic, and sinapic acids [50]. These acids are then converted to the corresponding alcohols and finally polymerize to form lignin [51]. The increase in phenolic acid contents and significant decrease in most carbohydrate contents in wounded scales demonstrated in our study indicate that the lignification process has been probably initiated. However, this assumption requires further examination.

A recently published article showed that the tissues of *Hippeastrum* (amaryllis) bulbs contain forty-eight alkaloids that may be useful in pharmacology [9]. Beyond the many alkaloids that are typical for the Amaryllidaceae family, other research shows that *Hippeastrum* has the ability to make a variety of flavonoids [10,11]. The results of our study indicate that stress caused by wounding and subsequent storage of wounded scales leads to a significant increase in the content of phenolic compounds in them. Since these compounds may also have medical significance, wounding and storing wounded amaryllis bulbs for several days may be one way to improve the quality of the pharmaceutical products made from them.

## 4. Materials and Methods

### 4.1. Plant Material

For the experiment, bulbs of *Hippeastrum* × *hybr*. Hort. (amaryllis) were used. The plant specimens were sourced from our internal cultivation program. The white bulb scales were cut into small pieces, about 2–4 mm square. One portion was frozen at −20 °C, while other samples of the cut scales were stored at ambient conditions, at temperature 18–22 °C in closed Petri dishes with the addition of small amounts of water to prevent them from drying. Then, after four days of storage, the red wounded scales were frozen. Subsequently, these scales and the white scales frozen prior to storage were freeze-dried and powdered, and the samples were used for the analysis of primary and secondary metabolites. Pictures were taken immediately after cutting the scales and four days of storage in daylight and/or darkness. The plant samples were freeze-dried for 48 h, during which the temperature of the condenser was 218 K (−55 °C) and the final pressure was 600 pA (0.06 mBar). This process was carried out in a laboratory freeze-dryer (Labconco, Kansas City, MO, USA).

### 4.2. Determination of Organic Acids and Amino Acids

Primary polar metabolites were extracted with a mixture of methanol/water (1:1, *v*/*v*), containing ribitol as an internal standard. The obtained supernatant was extracted with chloroform to remove lipid compounds, and the upper layer (methanol/water fraction) was dried in a speed vacuum rotary evaporator (JWElectronic, Warsaw, Poland). The compounds in dry residues were derivatized in two steps using *O*-methoxyamine hydrochloride in pyridine and a mixture of *N*-methyl-*N*-(trimethyl-silyl)-trifluoroacetamide in pyridine (both chemicals were from Sigma-Aldrich, Merck, Burlington, MA, USA). All further details of the method of the analysis have been described previously [52,53]. The identification and quantitative analyses of metabolites was confirmed by gas chromatography coupled with mass spectrometry (GCMS-QP2010 Plus, Shimadzu, Japan). Polar metabolites were identified by comparing the retention indices and mass spectra collected in the NIST 05 library (Shimadzu, Kyoto, Japan) and the internal collection of mass spectra obtained for the original standards purchased from SIGMA [54].

### 4.3. Determination of Soluble Carbohydrates

Soluble carbohydrates were extracted with a mixture of ethanol/water (1:1, *v*/*v*), containing xylitol as an internal standard, according to the method earlier described [55]. The carbohydrates were derivatized in a mixture of 1-(trimethylsilyl)imidazole in pyridine and then were separated on the GC capillary column ZEBRON ZB-1 (length, 15 m; diameter 0.25 mm; film thickness 0.10 μm; 100% dimethylpolysiloxane (Phenomenex, Torrance, CA, USA)).

### 4.4. Determination of Flavonoids and Phenolic Acids

The content of phenolic acids and flavonoids was determined according to the method described in detail by Dębski et al. [56]. Briefly, crude flavonoid extracts were obtained by stirring samples overnight with a mixture of methanol, water, and formic acid. Then, the free forms of phenolic acids and flavonoids were isolated from crude extracts with diethyl ether. Next, after the free forms were isolated, esters present in the extracts were hydrolyzed with 4 M NaOH and extracted with diethyl ether. Next, the glycosides were hydrolyzed with 6 M HCl and obtained free forms were extracted with diethyl ether. The free compounds and compounds released from bound forms were dissolved in methanol, centrifuged and subjected to analysis on an HPLC–MS/MS system (QTRAP 5500 ion trap mass spectrometer, AB SCIEX, Vaughan, ON, Canada) equipped with a HALO C18 column (2.7 μm particles, 0.5 × 50 mm, Eksigent, Vaughan, ON, Canada). The contents of phenolic compounds obtained by alkaline and acid hydrolysis were presented as their free, ester, and glycosidic forms or as a total of the all forms.

### 4.5. Determination of Anthocyanins

The extraction of anthocyanins and determination of their content was carried out using a method described in detail by Wiczkowski et al. [57]. Briefly, anthocyanins were extracted with 0.4% trifluoroacetic acid in methanol by vortexing and sonication, and the obtained extracts were centrifuged, and the supernatants were combined. The analyses were carried out using an LC-200 Eksigent HPLC system coupled with a Triple TOF 5600+ mass spectrometer (AB SCIEX, Vaughan, ON, Canada). Chromatographic separation of anthocyanins was carried out on the HALO C18 column (2.7 μm, 100 × 0.5 mm, Eksigent, Vaughan, ON, Canada) with a solvent gradient system consisting of solvent A (0.95% formic acid aqueous solution) and solvent B (0.95% formic acid in acetonitrile). Identification of the anthocyanins was based on a comparison of their retention time and MS/MS fragmentation spectrum (m/z values) with data of standards analysis and the published, or/and on the interpretation of the fragmentation spectrum obtained. Further details on the method of anthocyanin analysis were described in the cited reference [57].

### 4.6. Statistical Evaluation of Results

Results of measurements were subjected to analysis of variance (ANOVA), followed by Duncan’s multiple range test. The results of three replicates of primary and secondary metabolites were used for statistical analysis. *p* values of <0.05 were considered to be statistically significant for means.

## 5. Conclusions

Mechanical wounding of the tissues of *Hippeastrum* × *hybridum* Hort. (amaryllis) bulbs and their subsequent storage has a significant impact on the levels of the compounds contained therein. The color of mechanically wounded and stored amaryllis scales is the result of a mixture of many colorful phenolic compounds: flavonoids, phenolic acids and anthocyanins. Likely, the significantly increased content of several anthocyanins and luteolin in the wounded and stored scales resulted in a more intense color. A large decrease in content of l-phenylalanine to trace amounts indicates the intensity of biosynthesis of these compounds. The increased content of 4-coumaric, ferulic and sinapic acids, which are substrates for the biosynthesis of the appropriate alcohols used for production of lignin, suggests that this process also occurs. In addition, the significant decline in glucose, galactose and sucrose levels observed in our study indicates vigorous respiratory processes. These processes are both a source of energy and various metabolites that may contribute to counteracting wound-related stress. Mechanical wounding of the bulbs and their subsequent storage for several days may be a simple way to improve the quality of natural medicines made from amaryllis bulbs. However, this requires further detailed studies, especially with regard to compounds with pharmacological significance, such as alkaloids.

## Figures and Tables

**Table 1:** Total phenolic acid content (μg/g DW ± SD), as well as their aglycones (A), esters (E), and glycosides (G) in scales of *Hippeastrum* (amaryllis) bulb after mechanical wounding (white) and after 4 days of storage under ambient light conditions (red). Tr, traces—below 0.01 μg/g DW. Means in the rows marked with the same letter do not differ at the significance level of *p* < 0.05 according to Duncan’s test.

	White			Red		
Ferulic acid						
Total		22.70 ± 0.55 ^b^			50.73 ± 1.16 ^a^	
A/E/G	0.58 ± 0.09 ^c^	22.03 ± 0.43 ^b^	0.09 ± 0.03 ^d^	1.27 ± 0.48 ^c^	49.08 ± 0.61 ^a^	0.38 ± 0.08 ^c^
4-Coumaric acid						
Total		0.70 ± 0.03 ^b^			3.77 ± 0.07 ^a^	
A/E/G	0.03 ± 0.01 ^d^	0.66 ± 0.03 ^c^	Tr	1.10 ± 0.01 ^b^	2.63 ± 0.05 ^a^	0.04 ± 0.01 ^d^
Protocatechuic acid						
Total		0.10 ± 0.02 ^b^			0.37 ± 0.05 ^a^	
A/E/G	Tr	Tr	0.07 ± 0.01 ^c^	0.06 ± 0.02 ^c^	0.15 ± 0.01 ^a^	0.16 ± 0.02 ^a^
Caffeic acid						
Total		2.31 ± 0.10 ^b^			11.88 ± 0.36 ^a^	
A/E/G	0.04 ± 0.01 ^e^	2.14 ± 0.08 ^b^	0.12 ± 0.02 ^d^	0.63 ± 0.01 ^c^	11.06 ± 0.35 ^a^	0.19 ± 0.01 ^d^
Caftaric acid						
Total		0.03 ± 0.01 ^a^			0.09 ± 0.03 ^a^	
A/E/G	Tr	0.02 ± 0.01 ^a^	Tr	0.07 ± 0.02 ^a^	0.02 ± 0.02 ^a^	Tr
Ellagic acid						
Total		1.77 ± 0.19 ^b^			4.36 ± 0.21 ^a^	
A/E/G	0.77 ± 0.01 ^b^	0.33 ± 0.11 ^cd^	0.67 ± 0.07 ^bc^	3.46 ± 0.20 ^a^	0.29 ± 0.01 ^d^	0.61 ± 0.01 ^c^
Synapic acid						
Total		1.06 ± 0.24 ^b^			2.25 ± 0.02 ^a^	
A/E/G	Tr	1.06 ± 0.24 ^b^	Tr	Tr	2.25 ± 0.02 ^a^	Tr

**Table 2:** Total contents of flavonoids (μg/g DW ± SD), as well as their aglycones (A), esters (E), and glycosides (G) in scales of *Hippeastrum* (amaryllis) bulb after mechanical wounding (white) and after 4 days of storage in ambient light conditions (red). Tr, traces—below 0.01 μg/g DW. Means in the rows marked with the same letter do not differ at the significance level of *p* < 0.05 according to Duncan’s test.

White				Red		
			Quercetin			
Total		0.02 ± 0.01 ^b^			0.36 ± 0.02 ^a^	
A/E/G	Tr	Tr	0.02 ± 0.01 ^b^	0.33 ± 0.01 ^a^	Tr	0.03 ± 0.01 ^b^
			Apigenin			
Total		Tr			0.05 ± 0.01	
A/E/G	Tr	Tr	Tr	0.05 ± 0.01	Tr	Tr
			Kaempferol			
Total		0.02 ± 0.01 ^b^			0.78 ± 0.09 ^a^	
A/E/G	Tr	Tr	0.02 ± 0.01 ^b^	0.70 ± 0.07 ^a^	0.07 ± 0.02 ^b^	Tr
			Luteolin			
Total		0.07 ± 0.03 ^b^			13.05 ± 1.64 ^a^	
A/E/G	0.03 ± 0.01 ^c^	Tr	0.04 ± 0.01 ^c^	12.43 ±1.62 ^a^	0.56 ± 0.03 ^b^	0.06 ± 0.01 ^c^
			Catechin			
Total		6.33 ± 1.54 ^a^			4.12 ± 0.55 ^a^	
A/E/G	0.23 ± 0.21 ^bc^	0.08 ± 0.02 ^c^	6.01 ± 1.31 ^a^	0.71 ± 0.39 ^bc^	0.27 ± 0.07 ^c^	3.14 ± 0.09 ^a^
			Naringenin			
Total		0.02 ± 0.01 ^b^			5.83 ± 0.28 ^a^	
A/E/G	0.02 ± 0.01 ^c^	Tr	Tr	5.70 ± 0.27 ^a^	0.12 ± 0.01 ^b^	Tr

**Table 3:** Anthocyanin content (μg/g DW ± SD) in scales of *Hippeastrum* (amaryllis) bulb after mechanical wounding (white) and after 4 days of storage under ambient light conditions (red). Tr—traces, below 0.01 μg/g DW. Means in the rows marked with the same letter do not differ at the significance level of *p* < 0.05 according to Duncan’s test.

Determined Anthocyanin	White	Red
Cyanidin	Tr	5.75 ± 0.11
Pelargonidin	0.02 ± 0.01 ^b^	2.66 ± 0.07 ^a^
Cyanidin 3-glucoside	0.31 ± 0.04 ^b^	1.14 ± 0.03 ^a^
Delphinidin 3-glucoside	Tr	0.42 ± 0.05
Pelargonidin 3-glucoside	Tr	1.20 ± 0.01
Cyanidin diglucoside	0.02 ± 0.01 ^b^	2.29 ± 0.01 ^a^
Peonidin diglucoside	Tr	1.54 ± 0.07
Delphinidin diglucoside	0.29 ± 0.01 ^b^	1.20 ± 0.05 ^a^

**Table 4:** Contents (mg/g DW ± SD) of organic acids, amino acids, carbohydrates, and other polar compounds in scales of *Hippeastrum* (amaryllis) bulb after mechanical wounding (white) and after 4 days of storage in ambient light conditions (red). Means in the rows marked with the same letter do not differ at the significance level of *p* < 0.05 according to Duncan’s test.

Determined Metabolite	White	Red
Organic acids		
Acetic acid	0.17 ± 0.02 ^a^	0.07 ± 0.01 ^b^
Succinic acid	0.08 ± 0.01 ^a^	0.09 ± 0.01 ^a^
Malic acid	3.27 ± 0.09 ^a^	4.57 ± 0.53 ^a^
Citric acid	6.68 ± 0.32 ^a^	5.42 ± 0.61 ^a^
Amino acids		
Valine	0.18 ± 0.02 ^a^	0.10 ± 0.01 ^b^
Alanine	0.08 ± 0.01 ^a^	0.08 ± 0.01 ^a^
Serine	0.45 ± 0.01 ^a^	0.19 ± 0.03 ^b^
Leucine	0.14 ± 0.01 ^a^	0.06 ± 0.01 ^b^
Isoleucine	0.23 ± 0.01 ^a^	0.09 ± 0.02 ^b^
Proline	0.12 ± 0.01 ^a^	0.06 ± 0.01 ^b^
Hydroxyproline	0.49 ± 0.12 ^a^	0.33 ± 0.07 ^a^
l-Phenylalanine	0.13 ± 0.03 ^a^	0.01 ± 0.01 ^b^
Aspargic acid	0.70 ± 0.03 ^a^	0.42 ± 0.06 ^b^
Glutamic acid	0.14 ± 0.09 ^a^	0.04 ± 0.02 ^a^
Asparagine	0.06 ± 0.01 ^a^	0.12 ± 0.02 ^a^
γ-Amino butyric acid (GABA)	0.38 ± 0.07 ^a^	0.13 ± 0.04 ^b^
Carbohydrates		
Fructose	17.48 ± 0.15 ^a^	16.28 ± 1.87 ^a^
Galactose	3.26 ± 0.29 ^a^	1.81 ± 0.22 ^b^
Glucose	17.69 ± 0.66 ^a^	7.81 ± 0.71 ^b^
*myo*-Inositol	0.20 ± 0.02 ^a^	0.22 ± 0.04 ^a^
Sucrose	27.66 ± 1.35 ^a^	13.69 ± 1.62 ^b^
1-Kestose	20.40 ± 0.35 ^a^	14.38 ± 0.36 ^b^
Others		
Glycerol	0.22 ± 0.04 ^a^	0.26 ± 0.06 ^a^
Phosphoric acid	3.42 ± 0.09 ^a^	5.06 ± 0.67 ^a^
Total polar compounds	103.63 ± 1.62 ^a^	71.29 ± 6.09 ^b^

## Data Availability

All data are presented in the manuscript.

## Abbreviations

The following abbreviations are used in this manuscript:

JA-Me	Jasmonic acid methyl ester; methyl jasmonate
l-phe	l-phenylalanine
SD	Standard deviation
DW	Dry weight
Tr	Traces
GABA	γ-Amino butyric acid
